# L-Cysteine-Derived H_2_S Promotes Microglia M2 Polarization via Activation of the AMPK Pathway in Hypoxia-Ischemic Neonatal Mice

**DOI:** 10.3389/fnmol.2019.00058

**Published:** 2019-03-11

**Authors:** Xin Zhou, Xili Chu, Danqing Xin, Tingting Li, Xuemei Bai, Jie Qiu, Hongtao Yuan, Dexiang Liu, Dachuan Wang, Zhen Wang

**Affiliations:** ^1^Department of Physiology, Shandong University School of Basic Medical Sciences, Jinan, China; ^2^Department of Spinal Surgery, Shandong Provincial Hospital Affiliated to Shandong University, Jinan, China; ^3^Department of Medical Psychology, Shandong University School of Basic Medical Sciences, Jinan, China

**Keywords:** H_2_S, hypoxia-ischemia, M2 microglia, AMPK, complement protein

## Abstract

We have reported previously that L-cysteine-derived hydrogen sulfide (H_2_S) demonstrates a remarkable neuroprotective effect against hypoxia-ischemic (HI) insult in neonatal animals. Here, we assessed some of the mechanisms of this protection as exerted by L-cysteine. Specifically, we examined the capacity for L-cysteine to stimulate microglial polarization of the M2 phenotype and its modulation of complement expression in response to HI in neonatal mice. L-cysteine treatment suppressed the production of inflammatory cytokines, while dramatically up-regulating levels of anti-inflammatory cytokines in the damaged cortex. This L-cysteine administration promoted the conversion of microglia from an inflammatory M1 to an anti-inflammatory M2 phenotype, an effect which was associated with inhibiting the p38 and/or JNK pro-inflammatory pathways, nuclear factor-κB activation and a decrease in HI-derived levels of the C1q, C3a and C3a complement receptor proteins. Notably, blockade of H_2_S-production clearly prevented L-cysteine-mediated M2 polarization and complement expression. L-cysteine also inhibited neuronal apoptosis as induced by conditioned media from activated M1 microglia *in vitro*. We also show that L-cysteine promoted AMP-activated protein kinase (AMPK) activation and the AMPK inhibitor abolished these anti-apoptotic and anti-inflammatory effects of L-cysteine. Taken together, our findings demonstrate that L-cysteine-derived H_2_S attenuated neuronal apoptosis after HI and suggest that these effects, in part, result from enhancing microglia M2 polarization and modulating complement expression via AMPK activation.

## Introduction

Hypoxic-ischemic brain injury is the leading cause of neonatal mortality and permanent neurological deficits in surviving infants. Following HI, there is an immediate inflammatory response which then results in brain injury (Hagberg et al., 2015; Li et al., 2017). This neuroinflammation is primarily due to microglial activation that is associated with releasing cytokines or devastating substances thus leading to secondary neuronal injury. Activated microglia have classic (M1) and alternative (M2) activation phenotypes. M1 microglia demonstrate harmful properties by releasing pro-inflammatory mediators, such as IL-1β, IL-6, and TNF-α. In contrast, the M2 phenotype exhibits protective and restorative functions by releasing neurotrophic factors, such as insulin-like growth factor, epidermal growth factor, and nerve growth factor, as well as other anti-inflammatory factors, such as TGF-β, arginase-1, and IL-10 (Tang and Le, 2016). Results from recent studies have revealed that the regulation of microglial phenotypes can shift in response to a number of CNS injuries and thus exert beneficial effects for neurological recovery. For example, Fingolimod exerts a neuroprotective effect in chronic cerebral hypoperfusion via promoting STAT3-mediated modulation of microglia toward M2 polarization (Qin et al., 2017) and post-stroke, administration of omega-3 polyunsaturated fatty acids potentiated microglial M2 polarization resulting in white matter repair (Jiang et al., 2016). Moreover, mediating microglial phenotypes following HI in the developing brain may contribute to the neuroprotective effects of sodium butyrate (Jaworska et al., 2017).

H_2_S has been recently identified as an important gasotransmitter that regulates various intracellular and extracellular processes (Wang, 2012). Within the CNS, endogenous synthesis of H_2_S occurs mainly from L-cysteine (L-Cys) and homocysteine through cystathionine-β-synthase (CBS), cystathionine-γ-lyase, and 3-mercaptopyruvate. In addition, sulfurtransferase has also been reported to contribute to cysteine aminotransferase (Kimura, 2014). H_2_S has potential therapeutic effects, as demonstrated within several organs following ischemic injury as well as in metabolic diseases, by inhibiting inflammation and oxidative stress (Yu et al., 2015; De Cicco et al., 2018). An important factor which regulates inflammatory responses is the AMPK, a sensor of energy stress. Activation of AMPK signal pathways has been demonstrated to promote M2 macrophage/microglia polarization, thereby inhibiting inflammation (Mounier et al., 2013). Moreover, H_2_S donors were found to suppress inflammation via activating AMPK to polarize microglia to the M2 phenotype (Zhou et al., 2014). Thus, we investigate whether AMPK pathway is involved in this process of L-Cys in the study.

L-Cys is a substrate of H_2_S synthesis. Endogenous H_2_S is primarily produced in the mitochondria or cytosol from a cysteine substrate (l-cysteine and l-homocysteine). H_2_S is synthesized in most cells and tissues catalysis by enzymes: cystathionine-β-synthase (CBS), cystathionine-γ-lyase (CSE), and cysteine aminotransferase (CAT)/3-mercaptopyruvate sulphurtransferase (3-MST). And protects neurons from oxidative stress and is believed to produce this effect by increasing H_2_S synthesis (Shibuya et al., 2013). Previous results from our laboratory have also suggested that L-Cys plays a neuroprotective role in neonatal mice subjected to HI by reducing microglial activation via H_2_S production (Liu et al., 2017; Xin et al., 2018). However, the issue of whether L-Cys could mediate a microglial M1/M2 phenotype shift via the AMPK pathway remains obscure. Therefore, in this report we investigate some of the possible mechanisms through which L-Cys suppresses neuroinflammation in neonatal HI injury.

## Materials and Methods

### Materials

All details of materials used in this study are shown in Table 1.

**Table 1:** PCR primers in the text.

Gene	Forward (5′→ 3′)	Reverse (5′→ 3′)
TGFβ	GGT CCT TGC CCT CTA CAA CC	CCA CGT AGT AGA CGA TGC GC
IL-10	GTT GCC AAG CCT TAT CG	CCG CAT CCT GAG GGT CT
IL-1β	AAG ATG AAG GGC TGC TTC CAA ACC	ATA CTG CCT GCC TGA AGC TCT TGT
CD86	TAA GCA AGG TCA CCC GAA AC	AGC AGC ATC ACA AGG AGG AG
C1q	AAT GAC GCT TGG CAA CGT GGT TAT C	ATG AGG AAT CCG CTG AAG ATG CTG
C3a	GCC TGT CCT CTG AGC TCT GG	AGT TCT TCC CAC TGT TTC TGG
C3aR1	TGT TGG TGG CTC GCA GAT	GCA ATG TCT TGG GGT TGA AA
β-actin	CTA TTG GCA ACG AGC GGT TCC	CAG CAC TGT GTT GGC ATA GAG G

### Mouse HI Model

The animal model utilized in this study was that based on the Rice–Vannucci model (Vannucci and Vannucci, 1997), with slight modifications as that described in a prior publication (Liu et al., 2017). In brief, on postnatal day 7 (P7), C57BL/6J mice of both sexes were anesthetized with isoflurane (2% vol of isoflurane for inducing and 0.8% vol of isoflurane for maintaining anesthesia) and the right carotid artery was permanently ligated with use of one 4-0 nylon monofilament suture as performed under a dissecting microscope. The ligated pups were placed in a hypoxia chamber (humidified 92% N_2_ + 8% O_2_) for 90 min to induce the hypoxic insult, followed by placement into a recovery chamber for 60 min where the temperature was maintained at 36°C. Sham controls were anesthetized with 2.5% isoflurane on P7 and the right common carotid artery was separated but not ligated.

All animal experiments were performed according to the International Guiding Principle for Animal Research as stipulated by the Council for International Organizations of Medical Sciences (CIOMS) and all procedures were approval by the Shandong University Animal Ethics Committee. Investigators working with the animal model received training according to regulations of the Institutional Animal Care and Use Committee Guidebook (IACUC).

### Animal Groups

Each pup of mice (of both sexes) was allocated into five groups randomly and received treatment as shown in Supplementary Figure S1. AOAA (H_2_S-producing enzyme non-specific inhibitor), and L-Cys were dissolved in saline and administered through an intraperitoneal (i.p.) injection. CC (an AMPK inhibitor) was initially dissolved in DMSO (not exceeding 1% of the total volume), then diluted with saline and administrated through an i.p. injection. L-Cys (5 mg/kg) was administered at 24, 48, and 72 h following HI insult. AOAA (5 mg/kg) or CC (20 mg/kg) were administrated at 30 min prior to L-Cys injection. The HI (alone) and Sham groups were injected with equal amounts of the saline vehicle as based upon body weight. At 30 min following their final L-Cys injection, the animals were euthanized for further analysis.

Administration routes and doses for L-Cys and AOAA was according to our previous studies (Liu et al., 2017; Xin et al., 2018), while that for CC were chosen according to a previous study from McCullough et al. (2005). It should be noted that the doses of AOAA or CC used produced no significant signs of neurotoxicity, such as edema and infarct (Supplementary Figure S2).

### Collection of Tissue and Immunohistochemistry and Immunofluorescence Preparation

At 3 days after HI, anesthetized mice were perfused with 4% paraformaldehyde (PFA - pH 7.4). The brain was removed and placed in the same fixative at 4°C for 1 day. Brains were sliced in coronal sections at 4 μm thickness with samples between -1.60 and -2.00 mm from the bregma selected for the immunofluorescence and immunohistochemistry staining.

Immunofluorescence analysis was conducted as described previously (Liu et al., 2017). In brief, brain slices were treated with the following primary antibodies at 1:100: NeuN (Abcam, Cambridge, MA, United States), Cleaved caspase-3 (Cell Signaling Technology, Boston, MA, United States), Iba-1 (GeneTex, Inc., Irvine, CA, United States), CD16 (Abcam, Cambridge, MA, United States) or CD206 (Abcam, Cambridge, MA, United States). The Magna Fire SP system and fluorescent microscopy (OLYMPUS-BX51) were utilized for microphotographic analysis. The number of Cleaved caspase-3/NeuN and CD16/Iba-1 and/or CD206/Iba-1 double-positive cells in 6 microscopic fields were randomly selected from the ipsilateral cortex for evaluation. The total number of double-positive cells within every section was expressed as the average value of the 6 images per section (*N* = 4 mice/group). The data of CD16^+^/Iba-1^+^ and CD206^+^/Iba-1^+^ were expressed as the percent of double-positive cells relative to Iba-1-positive cells.

Immunohistochemistry analysis was conducted as described previously (Wang et al., 2012). In brief, each brain slice was incubated at 4^°^C overnight with anti-C1q (1:100) followed by secondary antibodies. Antibody binding analysis was performed with use of the DAB kit and each slide was then evaluated microscopically using the above mentioned Magna Fire SP system. The C1q^+^ cells within the infarct’s core region of cortex (*N* = 6 mice/group) were counted within 3 microscopic fields (×200 magnification). The number of C1q^+^ cells in each slice was expressed as the average value of 3 images per slice and this calculated value was then expressed as the percent of C1q^+^ cells relative to the Sham group.

### Western Blot Analysis

The ipsilateral cortex was extracted and frozen at -120°C. For immunoblots, the tissue was weighed upon the ice. After homogenization within RIPA buffer containing protease/ phosphatase inhibitors and PMSF, the tissue was centrifuged at 13800 × *g*/10 min at 4^°^C. The resultant supernatants were removed and 5X of loading buffer was added. Total concentrations of protein were quantified with use of the BCA Protein Assay Kits. Equivalent numbers of proteins were run on SDS-PAGE gels after being diluted with 5× loading buffer. Proteins were first electrophoresed for 30 min at 80 V, changed to 120 V for a minimum of 60 min and then transferred to PVDF membranes at 300 mA (1 h) utilizing a one wet transfer system. Membranes were blocked for 1 h and then maintained at 4°C overnight with the following primary antibodies: β-actin (Zhongshan Golden Bridge Biotechnology, Beijing, China) (1:1000), pNF-κB (Cell Signaling Technology, Boston, MA, United States) (1:500), NF-κB (Proteintech Group, Rosemont, IL, United States) (1:1000), p-p38 (Cell Signaling Technology, Boston, MA, United States) (1:1000), p38 (Proteintech Group, Rosemont, IL, United States) (1:1000), p-JNK (Santa Cruz Biotechnology, Santa Cruz, CA, United States) (1:500), JNK (Santa Cruz Biotechnology, SantaCruz, CA, United States) (1:500), p-IκBα (Proteintech Group, Rosemont, IL, United States) (1:500), IκBα (Proteintech Group, Rosemont, IL, United States) (1:1000), p-AMPK (Cell Signaling Technology, Boston, MA, United States) (1:1000) and AMPK (Cell Signaling Technology, Boston, MA, United States) (1:1000). The PVDF membranes were incubated with secondary antibodies at RT for 1 h. ECL kit reagents (MILLIPORE, United States) were used to develop the chemiluminescent signal, which was detected with use of the Tanon Imaging System (Tanon-4600).

### Reverse Transcription-PCR

The Ultrapure RNA Kit was used to extract total RNA from the ipsilateral cortex which had been frozen at -120°C. Reverse transcription was then conducted using the Revert Aid First Strand cDNA Synthesis Kit. PCR with specific primers were subsequently used to amplify the cDNA for β-actin, CD86 as well as IL-1β (Table 2). Electrophoresis was used to separate reaction products upon 1.2% of agarose/TAE gel containing 0.1% of GoldView (vol/vol). The reaction products were run for 30–40 min at 90 V and the GelDocXR System was used to capture the image. Image-Pro Plus 6.0 software was used for determining band intensities and each value was normalized to β-actin.

**Table 2:** Reagents in the text.

Reagents	Number	Company
L-Cysteine	C1276-10G	Sigma-Aldrich (St. Louis, MO, United States)
AOAA	A2129-10G	Sigma-Aldrich
TTC	T8877-10G	Sigma-Aldrich
Compound C	P5499-5MG	Sigma-Aldrich
Phospho-NF-κB p65(Ser536) (93H1)	#3033	Cell Signaling
Phospho-AMPKα (Thr172)	#2535	Cell Signaling
AMPKα	#5831	Cell Signaling
Phospho-p38 MAPK (Thr180/Tyr182)(3D7)	#9215	Cell Signaling
Phospho- IkBa (Ser32)	#2859	Cell Signaling (Beverly, MA, United States)
Iba-1	GTX632426	GeneTex, Inc. (Irvine, CA, United States)
Anti-NeuN	ab104224	Abcam (Cambridge, MA, United States)
Anti-C1q	Ab71089	Abcam
GFAP	60190-1-Ig	Proteintech Group (Rosemont, IL, United States)
Anti-IkBa	10268-1-AP	Proteintech Group
Anti-p38 MAPK	14064-1-AP	Proteintech Group
Anti-P65	10745-1-AP	Proteintech Group
Phospho-JNK MAPK	Sc-6254	Santa Cruz Biotechnology (Santa Cruz, CA, United States)
JNK MAPK	Sc-7345	Santa Cruz Biotechnology
Anti-β-actin	TA-09	Zhongshan Golden Bridge Biotechnology (Beijing, China)
Rhodamine (TRITC)-conjugated goat anti-rabbit IgG	SA00007-2	Proteintech Group
Fluorescein (FITC)-conjugated affinipure goat anti-mouse IgG	SA00003-1	Proteintech Group
Peroxidase-conjugated goat anti-rabbit IgG	ZB-2301	Zhongshan Golden Bridge Biotechnology
Peroxidase-conjugated goat mouse IgG	ZB-2305	Zhongshan Golden Bridge Biotechnology
Enhanced chemiluminescence and PVDF membranes	IPVH00010	Millipore Corporation (Billerica, MA, United States)
PhosSTOP phosphatase inhibitor	P1082	Roche Diagnostics Gmbh (Indianapolis, IN, United States)
RIPA	P0013B	Beyotime Institute of Biotechnology (Jiangsu, China)
PMSF	ST506-2	Beyotime Institute of Biotechnology
5× loading buffer	P0015L	Beyotime Institute of Biotechnology
ReverTra Ace Qpcr RT Kit	FSQ-101	TOYOBO (Kita-ku, Osaka, Japan)
TRIzon reagent	01761/20114-1	CWBIO (Haimen, Jiangsu, China)
BCA protein assay kit	CW0014S	CWBIO
GolView	G8140	Beijing Solarbio Science & Technology Company (Beijing, China)
TUNEL apoptosis detection kit	KGA7051-B	KeyGen Bio Tech Co. (Nanjing, Jiangsu, China)
Immobilon Western Chemiluminescent HRP Substrate	WBKLS0100	Millipore Corporation

### Brain Water Content

As previously described, brains were removed at 24 h after HI and bisected to generate two hemispheres (ipsilateral and contralateral to the injury) which were then immediately weighed. The hemispheres were placed in an oven (105°C) to dry for 48 h and then weighed again. The brain water content was calculated using the following formula:

Brainwatercontent% = [(wetweight - dryweight)/wetweight] × 100%

### Infarct Ratio Measurement

Brains were rapidly removed at 3 days post-HI and hardened in a refrigerator (-20°C) for 20 min and for slicing. And then divided into 4 coronal sections, each 1mm thick. Each section was incubated with 2% TTC at 37°C for 20 min. The software, Image-Pro Plus 6.0 was used for summation of the volume of each section. An infarction percent (rate of infarct) was calculated by dividing infarct volume by total section volume.

### Analysis of Brain Microglia/Macrophages

Single cells were prepared as previously described [12]. Briefly, at 3 days after HI insult, the right cerebral cortex tissue from each group was placed in a pre-cooled Hank’s balanced salt solution (HBSS). The tissue was cut into approximately 1 mm^3^ sections and digested with 10 ml digestive solution at 37°C for 40 min. The digested tissue was then crushed to generate a cell suspension, filtered through a 70 μm sieve and centrifuged at 420 × *g* for 10 min. The resultant pellet was resuspended with 4 mL 40% Percoll solution (GE Health Care BioSciences). Then, 4 mL 70% Percoll solution was slowly added to the lower cell suspension using a syringe and centrifuged at RT at 500 × *g* for 20 min. One portion of 10× PBS was mixed with 9 parts of Percoll stock solution for preparing an isotonic suspension of Percoll considered as a 100% suspension of Percoll. The 100% Percoll was diluted with 1× PBS to achieve an expected density of Percoll separation solution for cell isolation. Cells were harvested from the interface of the different concentrations of Percoll solution and rinsed once with PBS containing 0.2% of BSA. The cells were stained with the following antibodies: anti-mouse CD11b-FITC or mouse CD45-APC for population evaluation of activated microglial cells/macrophages (CD11b^+^/CD45^high^ cells). A FACS flow cytometer C6 (BD Biosciences) was adopted to perform the flow cytometric analysis.

### Primary Neuron Cultures and Microglia Conditioned Media Treatments

Cells from the microglia-like cell line-BV2 were seeded into 6-well plates and incubated overnight. The BV2 cells were treated with/without L-Cys for 1 h followed by LPS for 24 h with DMEM:F12 media. This media (CM) was then collected and stored at -80°C until future use.

Primary cultures of isolated cortical cells were prepared and maintained as described previously (Wang et al., 2012). In brief, the cortex of P1 mice was isolated and placed in 24-well plates that were pre-coated with the poly-L-lysine in neurobasal medium without serum along with one B27 supplement. The cells were all allowed to differentiation for 7 days, at which time the neuronal medium was removed and substituted with that of the CM from BV2 cells. To analyze the effects of CM on cell apoptosis, neurons were cultured with CM for 24 h. The controls received basal BV2 cell media.

### TUNEL Staining

Cells were naturally dried in a dyeing cylinder containing 4% PFA fixative, fixed for 20–30 min at RT and then immersed in permeable solution (1% Triton X-100) for 3–5 min to promote permeability. After three rinses with PBS cells were immersed in sealing solution (3% H_2_O_2_) for 10 min. DNaseI solution (100 μl) was added to each sample, treated at RT for 10–30 min and then rinsed three times with PBS. Each sample was then treated with 50 μl TdT enzyme reaction liquid, maintained in a warm box for 60 min. Then each sample covered with 50 μl Streptavidin – TRITC working solution at 37°C for 30 min. Fluorescence microscopy (excitation wavelength 450–500 nm, emission wavelength 515–565 nm) was determined with use of fluorescence microscopy after three rinses with PBS.

### Statistical Analysis

A minimum of 3 ipsilateral cortex from 3 mice were used in the experiments for analyses of PCR, flow cytometry, Western blot, TUNEL staining, immunohistochemistry and immunofluorescence on brain section, which were independently repeated three times. Data were analyzed with use of the SPSS software program (version 20, IBM, New York, NY, United States). All values are expressed as the mean ± standard deviation (SD). Data were analyzed with one-way ANOVA and the Tukey test for *post hoc* comparisons. A *p* < 0.05 was required for results to be considered statistically significant.

## Results

### L-Cys Promoted a M1 to M2 Microglia Polarization Shift at 72 h Post-HI

**FIGURE 1:**
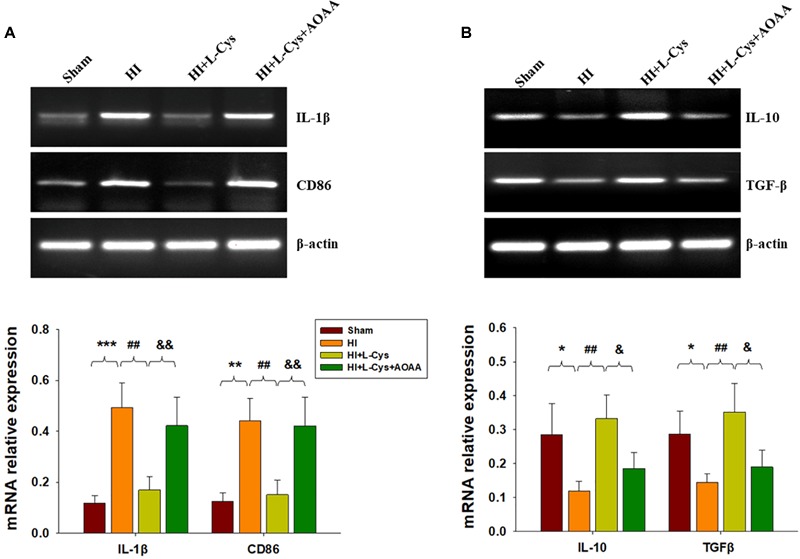
L-Cys inhibited HI-induced neuro-inflammation at 72 h post-HI. **(A,B)** Representative RT-PCR photographs showing mRNA levels of IL-1β, CD86, IL-10 and TGFβ within the ipsilateral cortex. Quantitative results of relative mRNA levels among the different groups. Values were normalized to β-actin. *N* = 4 mice/group. Values represent the mean ± SD, ^∗^*p* < 0.05, ^∗∗^*p* < 0.01, ^∗∗∗^*p* < 0.001 HI vs. Sham; ^##^*p* < 0.01 HI+L-Cys vs. HI; ^&^*p* < 0.05, ^&&^*p* < 0.01 HI+L-Cys + AOAA vs. HI+ L-Cys.

Gene expressions of M1 (IL-1β and CD68) and M2 (IL-10 and TGF-β) markers in the ipsilateral cortex were determined with use of RT-PCR. HI induced increased levels of IL-1β (*p* < 0.001) and CD86 (*p* < 0.01) mRNA as compared with that observed in the Sham group, while these HI-induced increases in pro-inflammatory cytokines were significantly suppressed (*p* < 0.01 for both) by L-Cys treatment (Figure 1A). In contrast, HI insult suppressed M2 marker (IL-10 and TGF-β) expressions as compared to the Sham group (*p* < 0.05 for both) and L-Cys significantly increased the gene expressions of these two anti-inflammatory cytokines (*p* < 0.05 for both).

**FIGURE 2:**
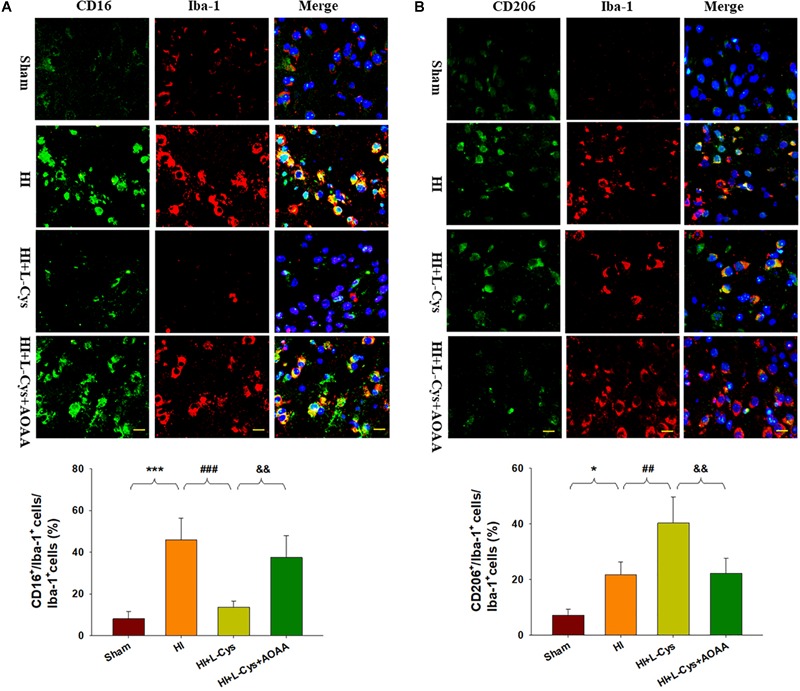
L-Cys inhibited HI-induced microglia M1 polarization and promoted microglia M2 polarization at 72 h post-HI. **(A)** Representative photographs of double immunofluorescent staining of CD16 (red), and Iba-1 (green) cells within the ipsilateral cortex. Scale bar = 20 μm. **(B)** Representative photographs of double immunofluorescent staining of CD206 (red) and Iba-1 (green) cells within the ipsilateral cortex. Scale bar = 20 μm. Quantification of CD16^+^/Iba-1^+^ and CD206^+^/Iba-1^+^ cells (*N* = 4 mice/group). Six images (20×) were randomly selected from each section per animal. Values represent the mean ± SD, ^∗^*p* < 0.05, ^∗∗∗^*p* < 0.001 HI vs. Sham; ^##^*p* < 0.01, ^###^*p* < 0.001 HI + L-Cys vs. HI; ^&&^*p* < 0.01 HI + L-Cys + AOAA vs. HI + L-Cys.

To further confirm these changes in M1/M2 polarization to L-Cys treatment, we next examined these responses using immunofluorescence analysis. Significantly increased numbers of CD16^+^/Iba1^+^ (*p* < 0.001) and CD206^+^/Iba1^+^ (*p* < 0.05) cells were obtained within the ipsilateral cortex of the HI as compared with the sham group (Figure 2). However, while L-Cys administration further increased the number of CD206^+^/Iba1^+^ cells (*p* < 0.01; Figure 2B), it reduced the number of CD16^+^/ Iba1^+^ cells (*p* < 0.001; Figure 2A). The effects of L-Cys on neuroinflammation and microglial polarization were reversed with use of AOAA (an inhibitor of the H_2_S producing enzyme) (Figure 1, 2).

### Effects of L-Cys on C1q, C3α and C3aR1 Expressions at 72 h Post-HI

**FIGURE 3:**
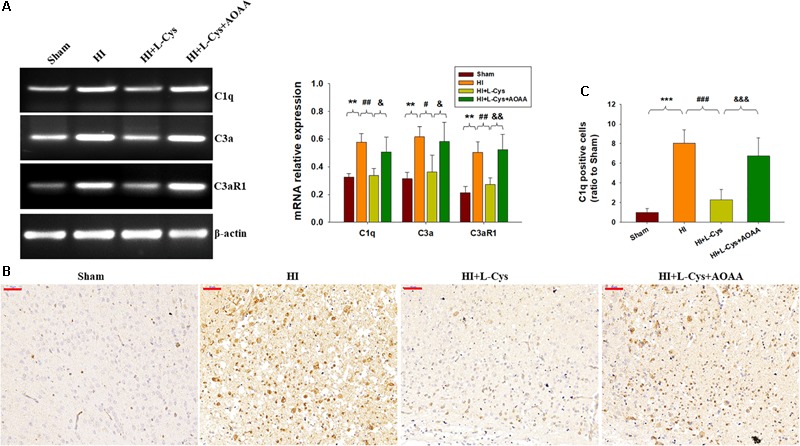
L-Cys suppressed HI-induced C1q, C3a, and C3aR1 expression in the ipsilateral cortex at 72 h post-HI. **(A)** Representative RT-PCR photographs showing mRNA levels of C1q, C3a, and C3aR1 within the ipsilateral cortex. Quantitative results of relative mRNA levels among the different groups. Values were normalized to β-actin. *N* = 4 mice/group. **(B)** Representative photographs of C1q staining within the ipsilateral cortex as taken at 72 h following HI. Scale bar = 50 μm. **(C)** Quantification of C1q^+^ cells. *N* = 6 mice/group. Values represent the mean ± SD, ^∗∗^*p* < 0.01, ^∗∗∗^*p* < 0.001 HI vs. Sham; #*p* < 0.05, ^##^*p* < 0.01, ^###^*p* < 0.001 HI + L-Cys vs. HI; ^&^*p* < 0.05, ^&&^*p* < 0.01, ^&&&^*p* < 0.001 HI + L-Cys + AOAA vs. HI + L-Cys.

As the complement system initiates inflammation and regulates antibody production, we next investigated the effects of L-Cys on levels of complement factors following HI. HI increased the expressions of C1q, C3α and C3aR1 (*p* < 0.01 for all) within the ipsilateral cortex as compared to that of the Sham group (Figure 3A). In response to L-Cys treatment the elevations of C1q, C3α, and C3aR1 were significantly attenuated (*p* < 0.01, *p* < 0.05, *p* < 0.01, respectively; Figure 3A). Further confirmation of these C1q findings were obtained from results of immunohistochemistry analysis (Figure 3B,C). When L-Cys administration was combined with AOAA, the effects of L-Cys on modulating C1q, C3α and C3aR1 expressions were compromised (Figure 3).

### L-Cys Suppressed NF-κB Activation at 72 h Post-HI

**FIGURE 4:**
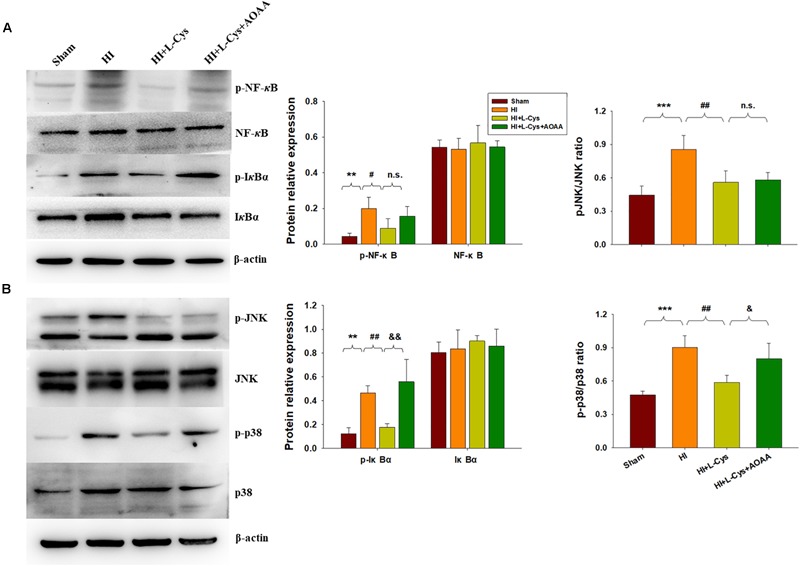
Effects of L-Cys on NF-κB activation, JNK and p38 phosphorylation in the ipsilateral cortex at 72 h post-HI. **(A)** Representative immunoblots are shown for phosphorylated NF-κB (p-NF-κB), total NF-κB, phosphorylated-IκBα (p-IκBα) and total IκBα as determined with western blotting analysis. Quantification of relative levels of p-NF-κB, NF-κB, p-IκBα and IκBα in each group. Values were normalized to β-actin. *N* = 4 mice/group. **(B)** Representative immunoblots of phosphorylated JNK (p-JNK), total JNK, phosphorylated-p38 (p-p38) and total p38 protein levels using western blotting analysis. Quantification of relative levels of p-JNK/JNK and p-p38/p38 in each group. *N* = 4 mice/group. Values represent the mean ± SD, ^∗∗^*p* < 0.01, ^∗∗∗^*p* < 0.001 HI vs. Sham; ^#^*p* < 0.05, ^##^*p* < 0.01, HI + L-Cys vs. HI; ^&^*p* < 0.05, ^&&^*p* < 0.01 HI + L-Cys + AOAA vs. HI + L-Cys.

The NF-κB pathway plays an important role in microglial inflammatory activation. Therefore, we investigated whether the NF-κB pathway would be involved in these anti-inflammatory effects of L-Cys. We found that the expressions of p-NF-κB (*p* < 0.01) and p-IκBα (*p* < 0.01) were up-regulated in the HI group as compared with that of the Sham group and L-Cys treatment significantly reduced these HI-induced expressions of p-NF-κB (*p* < 0.05) and p-IκBα (*p* < 0.01) (Figure 4A). When L-Cys administration was combined with AOAA, the effects of L-Cys on NF-κB activation were blunted.

### L-Cys Suppressed JNK and p38 Activation at 72 h Post-HI

The MAPK pathway represents another important transcription factor involved with the regulation of gene expressions associated with the M1 phenotype. With regard to this pathway, HI exposure increased the expressions of p-JNK (*p* < 0.001) and p-p38 (*p* < 0.001) within the ipsilateral cortex at 72 h following injury. L-Cys treatment significantly reduced these HI-induced increased expressions of p-p38 (*p* < 0.01) and p-JNK (*p* < 0.01) as compared with that obtained in the HI group. A co-treatment of L-Cys with AOAA reversed the effect of L-Cys on p-p38, but not p-JNK, following HI insult (Figure 4B).

### L-Cys Promotes M2 Polarization Through AMPK Activation in the HI Damaged Brain

**FIGURE 5:**
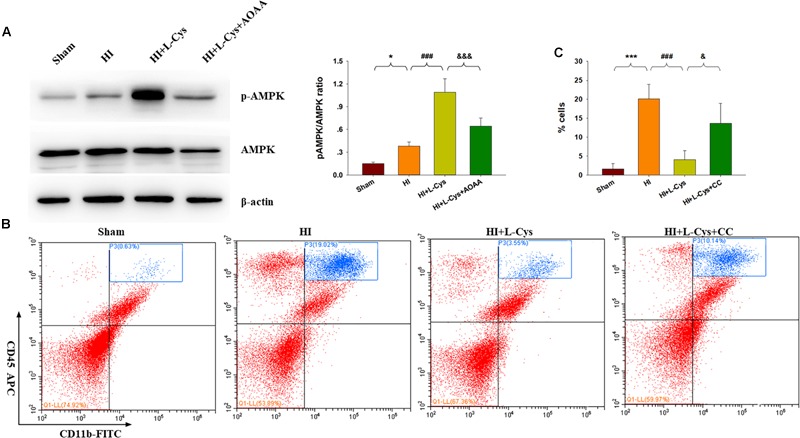
AMPK activation is involved in the anti-inflammatory effects of L-Cys treatment following HI. **(A)** Representative immunoblots of phosphorylated AMPK (p-AMPK), AMPK and β-actin protein levels. Quantification of relative levels of p-AMPK/AMPK in each group. *N* = 4 mice/group. **(B)** Representative flow cytometric plots of CD11b^+^/CD45^high^ cells within the ipsilateral cortex. **(C)** Quantification of CD11b^+^/CD45^high^ cell numbers. *N* = 4 mice/group. ^∗^*p* < 0.05, ^∗∗∗^*p* < 0.001 HI vs. Sham; ^###^*p* < 0.001 HI + L-Cys vs. HI; ^&^*p* < 0.05, ^&&&^*p* < 0.001 HI + L-Cys + AOAA/CC vs. HI + L-Cys.

H_2_S donors were found to suppress inflammation via activating AMPK to polarize microglia to the M2 phenotype. In this experiment, we investigated whether AMPK is involved in L-Cys-promotion of M2 microglia. Results from Western blot assays revealed that p-AMPK expression was significantly increased in the ipsilateral cortex of the HI as compared to that of Sham group (*p* < 0.05) and L-Cys treatment further upregulated this p-AMPK expression (*p* < 0.001; Figure 5A). Moreover, the capacity for L-Cys to attenuate the accumulation of CD11b^+^/CD45^high^ cells within the ipsilateral cortex was blocked by the AMPK inhibitor CC, when administered prior to L-Cys in the immature brain following HI (*p* < 0.05; Figure 5B).

**FIGURE 6:**
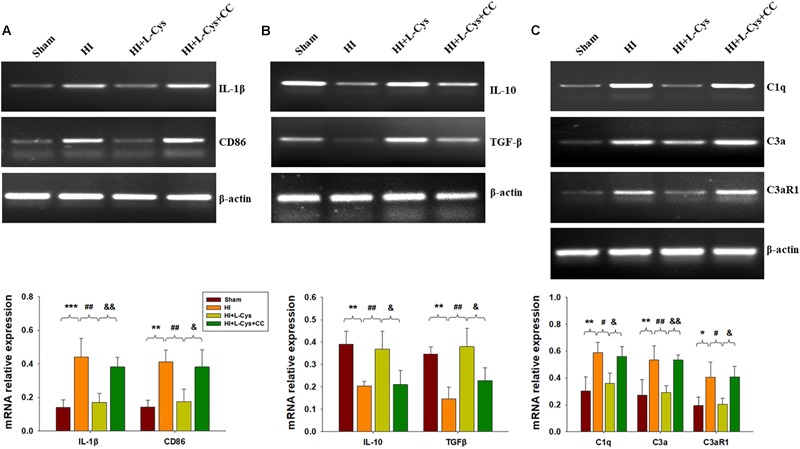
AMPK activation is involved in L-Cys- mediated M2 polarization and complement expression. Representative RT-PCR photographs showing mRNA levels of IL-1β and CD86 **(A)**, IL-10 and TGFβ **(B)**, and C1q, C3a, and C3aR1 **(C)** within the ipsilateral cortex. Quantitative results of relative mRNA levels among the different groups. Values were normalized to β-actin. *N* = 4 mice/group. Values represent the mean ± SD, ^∗^*p* < 0.05, ^∗∗^*p* < 0.01, ^∗∗∗^*p* < 0.001 HI VS Sham; ^#^*p* < 0.05, ^##^*p* < 0.01 HI + L-Cys vs. HI; ^&^*p* < 0.05, ^&&^*p* < 0.01 HI + L-Cys + CC vs. HI + L-Cys.

Prior treatment with CC reversed the L-Cys-mediated an up-regulation of M2 markers (IL-10 and TGF-β; Figure 6A) and a down-regulation of classical M1 marker (IL-1β and CD86; Figure 6B). In addition, this CC treatment also reversed the L-Cys-mediated C1q, C3α and C3aR1 expressions within the ipsilateral cortex following HI injury (Figure 6C).

### L-Cys Attenuates Microglia-Mediated Neurotoxicity via AMPK Activation

**FIGURE 7:**
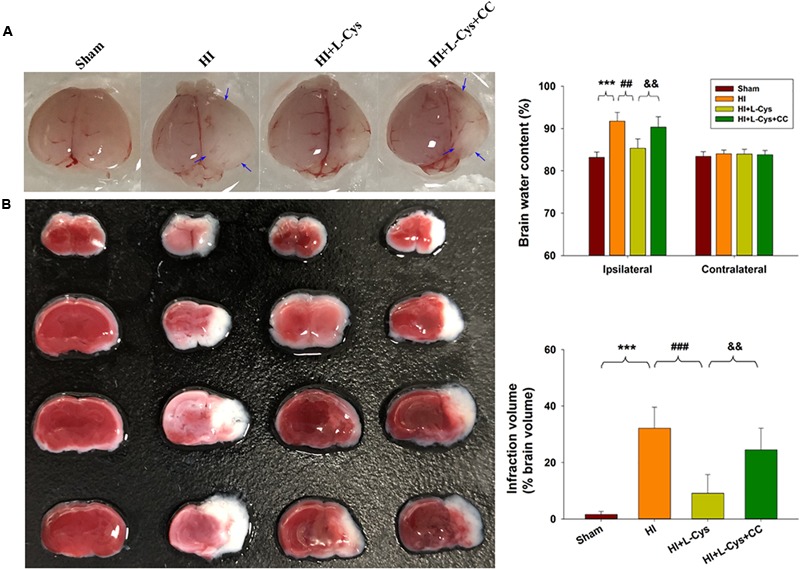
L-Cys attenuates HI-induced brain injury via AMPK activation. **(A)** Representative brain photographs as determined at 72 h following HI. Arrows indicate sites of significant edema. Brain water content was determined at 72 h following HI insult, *N* = 5 mice/group. **(B)** Representative samples stained with TTC. Infarct volume (white area) was quantified. *N* = 5 mice/group. Values represent the mean ± SD, ^∗∗∗^*p* < 0.001 HI vs. Sham; ^##^*p* < 0.01, ^###^*p* < 0.001 HI + L-Cys vs. HI; ^&&^*p* < 0.01 HI + L-Cys + CC vs. HI + L-Cys.

**FIGURE 8:**
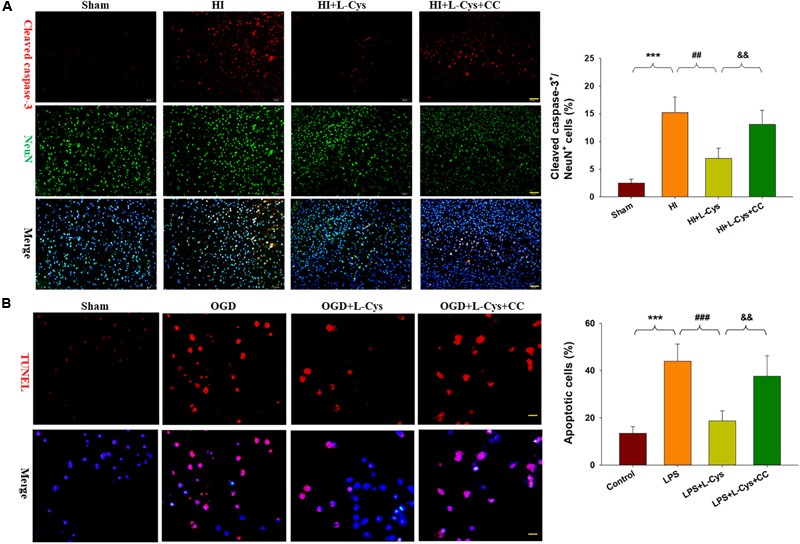
L-Cys attenuates microglia-mediated neurotoxicity via AMPK activation. **(A)** Representative photographs of double immunofluorescent staining of active caspase-3 (red) and NeuN (green) within the ipsilateral cortex. Scale bar = 50 μm. Quantification of active caspase-3/NeuN-positive cells. *N* = 3 mice/group. Six images (20×) were randomly selected from each section per animal. **(B)** Effects of different conditioned media prepared from microglia-like cell line-BV2 cells on the survival of neurons as determined with TUNEL staining (red). Scale bar = 20 μm. *N* = 4 wells/group. Values represent the mean ± SD, ^∗∗∗^*p* < 0.001 HI vs. Sham; ^##^*p* < 0.01, ^###^*p* < 0.001 HI + L-Cys vs. HI; ^&&^*p* < 0.01 HI + L-Cys + CC vs. HI + L-Cys.

We also found that L-Cys remarkably protected against acute injury induced by HI, which confirmed previous observations within our laboratory (Liu et al., 2017). And, administration of the AMPK inhibitor, CC, prior to L-Cys in immature mice significantly blunted the L-Cys effects on edema (*p* < 0.01; Figure 7), infarction (*p* < 0.001; Figure 7) and neuronal apoptosis (*p* < 0.001; Figure 8A).

**FIGURE 9:**
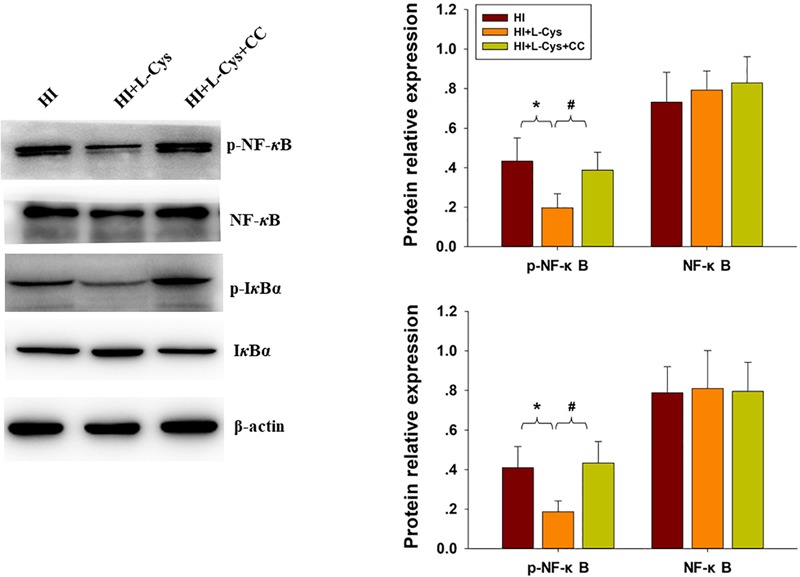
Blocking AMPK activation reversed L-Cys effects on NF-κB activation following HI insult. Representative immunoblots are shown for phosphorylated NF-κB (p-NF-κB), total NF-κB, phosphorylated-IκBα (p-IκBα) and total IκBα as determined with western blotting analysis. Quantification of relative levels of p-NF-κB, NF-κB, p-IκBα and IκBα in each group. Values were normalized to β-actin. *N* = 4 mice/group. Values represent the mean ± SD, ^∗^*p* < 0.05 HI + L-Cys vs. HI; ^#^*p* < 0.05 HI + L-Cys + CC vs. HI + L-Cys.

Finally, we used conditioned medium from microglial cultures to observe whether L-Cys could attenuate microglia-mediated neurotoxicity as demonstrated *in vitro*. CM from LPS-treated microglia cells led to significant neuronal apoptosis (*p* < 0.001). However, when CM from LPS was combined with L-Cys (1 μM) there was a significant decrease in apoptosis within these primary neurons (*p* < 0.01); and the AMPK inhibitor, CC (5 μM), significantly blunted these effects of L-Cys on neuronal apoptosis (*p* < 0.05; Figure 8B). We found that the AMPK inhibitor, CC significantly reversed the L-Cys-reduced expressions of p-NF-κB (*p* < 0.05) and p-IκBα (*p* < 0.05) within the ipsilateral cortex following HI insult (Figure 9).

## Discussion

Results from numerous studies have shown the protective effects of H_2_S upon a diverse array of pathological conditions. Our data are in general agreement with this L-Cys -derived H_2_S reduction in HI-related neuronal death. And, here we expand upon these findings by demonstrating that this effect is achieved by enhancing microglia M2 polarization and decreasing C1q, C3a and C3aR1 expression via AMPK activation. In addition, we also found that the NF-κB and MAPK pathways also play roles in these anti-inflammatory effects of L-Cys in the immature brain following HI.

Microglia-mediated neuroinflammation is involved in hypoxia-ischemia as well as in other diseases. Within the damaged CNS, a switch in microglial polarization from pro-inflammation M1 phenotype to anti-inflammatory/repair M2 phenotype exert different functions. For example, M1-polarized microglia release various pro-inflammatory cytokines or mediators, which produce neuronal cell damage, while M2-polarized microglia generally result in responses which suppress inflammation and promote tissue remodeling via various anti-inflammatory cytokines and neurotrophic factors. The M1/M2 phenotype shift has been shown in animal models of neonatal HI, traumatic brain injury and neurodegenerative disease (Hu et al., 2012; Song and Suk, 2017; Wen et al., 2018). We have reported that H_2_S remarkably attenuates pro-inflammatory cytokine release and provides neuroprotective roles in neonatal HI mice (Xin et al., 2018). In addition, H_2_S suppresses LPS-induced M1 gene expression, whereas M2 gene expression was enhanced in microglia, as demonstrated *in vivo* and *in vitro* (Zhou et al., 2014). Consistent with these previous findings, here we show that L-Cys treatment not only decreased expression of the HI-induced M1 phenotype, but also increased M2 microglia polarization within the ipsilateral hemisphere of HI mice. And, L-Cys significantly inhibited activated M1 microglia-induced neuronal death. These data suggested that the anti-neurotoxic effects of L-Cys may, in part, be due to regulating this M1 to M2 microglia shift.

Accumulating evidence has been presented which indicates that the complement system is a component of inflammatory responses and is involved in CNS injury (Arumugam et al., 2006). In support of these findings, C1q, which is significantly up-regulated in the brain following insult, produces pro-inflammatory mediators including C3a and C5a through classical complement pathway activation and deletion of the C1q gene exerts long-lasting neuroprotection against HI in neonatal mice (Ten et al., 2005). The complement component, C9, significantly augments brain injury when administered at 24 h post-HI insult in newborn animals (Imm et al., 2002). Conversely, administration of the C3a peptide attenuated HI-induced injury in the immature brain, which the protective effects of C3a were independent of the suppressed expression of proinflammatory cytokines (Jarlestedt et al., 2013; Moran et al., 2017). In the current study, we demonstrated that elevations in C1q, C3a and C3aR1 mRNA levels in the ipsilateral hemisphere were associated with inflammatory responses and L-Cys treatment inhibited these HI-induced elevated expressions, suggesting that inhibition of these complement effectors plays a role in H_2_S neuroprotection. Microglia-mediated synapse loss has been implicated in the pathophysiology of Alzheimer’s disease (Hong et al., 2016) and the age-dependent increases in C1q which are localized in or around synapses likely play a role in the cognitive decline associated with normal brain aging (Stephan et al., 2013). Neonatal ventral hippocampal lesions have been shown to increase microglial reactivity and expression of complement molecules, which led to synaptic reorganization and behavioral deficits (Hui et al., 2018). We have previously reported that L-Cys treatment improved HI-induced cognitive deficits, effects which were associated with a significant attenuation in synaptic loss (Xin et al., 2018). Based upon these results we speculated that suppressing complement effector activation with L-Cys contributes to modulating synapses integrity following HI.

Notably, the role of inhibiting C3a and C3aR1 expression after HI in this study is in contrast to the beneficial effects of C3a–C3a receptor signaling as reported previously (Jarlestedt et al., 2013; Moran et al., 2017). One possible explanation for these findings is that the neuroprotective property of L-Cys in HI insult may be regulated by additional mechanisms that may be parallel to or independent of suppressed complement effectors. For example, H_2_S can contribute to an anti-inflammatory, anti-apoptotic and antioxidative functions in various CNS diseases, such as cerebral ischemia, Alzheimer disease and Parkinson’s disease (Shefa et al., 2018).

AMPK phosphorylation has also been demonstrated to inhibit inflammation responses and promote M1–M2 shifts (Zhou et al., 2014; Li et al., 2018). In the current study, L-Cys robustly increased AMPK activation in the ipsilateral cortex, results which are consistent with a previous report showing that H_2_S activates AMPK to promote microglia to M2 polarization following LPS stimulated (Zhou et al., 2014). Moreover, prior treatment with an AMPK inhibitor blunts these effects of L-Cys in enhancing M2 gene expression and attenuating neuronal apoptosis following HI, suggesting that the anti-apoptotic capacity of L-Cys after HI may be regulated through the AMPK pathway.

Another regulator of inflammatory responses is that of NF-κB signaling (Perkins, 2007). Under inflammatory conditions, NF-κB translocates to the nucleus, which then activates mRNA expression of target genes. In the current study, we demonstrate that HI insult in neonatal mice activated NF-κB signaling, as revealed by an up-regulation in the phosphorylation of p65 and IκBα in the ipsilateral cortex. L-Cys robustly decreased this phosphorylation of p65 and IκBα at 72 h following HI injury, an effect which was associated with a reduction in NF-κB-activated inflammatory genes. In accordance with our results, H_2_S has been reported to exhibit anti-inflammatory effects via NF-κB pathways (Oh et al., 2006; Huang et al., 2016). Interestingly, in these previous studies, it was shown that NF-κB activation following HI in neonatal rats exerted a biphasic effect. Such a biphasic effect may provide one explanation for the inconsistencies reported in the role of NF-κB activation within neonatal animals subjected to HI as different times of sampling post-HI may produce different results. For example, inhibition of NF-κB activation in the early phases after HI (0.5–6 h) results in a neuroprotective effect, while inhibition of NF-κB activation in later phase (24 h) contributes to neonatal HI-induced brain damage, which is associated with a reduced expression of anti-apoptotic factors (Nijboer et al., 2008).

In addition to the NF-κB pathway, a critical role of the MAPK pathway family (p38, JNK and ERK) in the regulation of inflammation has also been reported (Karin, 2004). In the present study, L-Cys treatment significantly suppressed phosphorylations of p38 and JNK within the ipsilateral cortex following HI and H_2_S has been demonstrated to exhibit anti-inflammatory effects via p38 and/or JNK MAPK pathways (Issa et al., 2013; Zhang et al., 2014). Taken together, these findings suggest that L-Cys can prevent microglia-mediated neuroinflammation by suppression of p38 and/or JNK MAPK pathways and an inhibition of the phosphorylation of NF-κB.

Our study has several limitations. First, neuron-microglia cocultures were not used to examine neuronal survival via microglial polarization following HI and microglia were only determined *in vitro* after exposure to LPS. Second, the role and underlying mechanisms regulating the effects of L-Cys on complement expression were not explored. Third, we did not investigate whether L-Cys affected long-term microglial responses after HI. Finally, levels of H_2_S following L-Cys treatment were not measured in the current study, as were done in a previous report (Xin et al., 2018). Further investigations will be required to address the above-mentioned points.

## Conclusion

**FIGURE 10:**
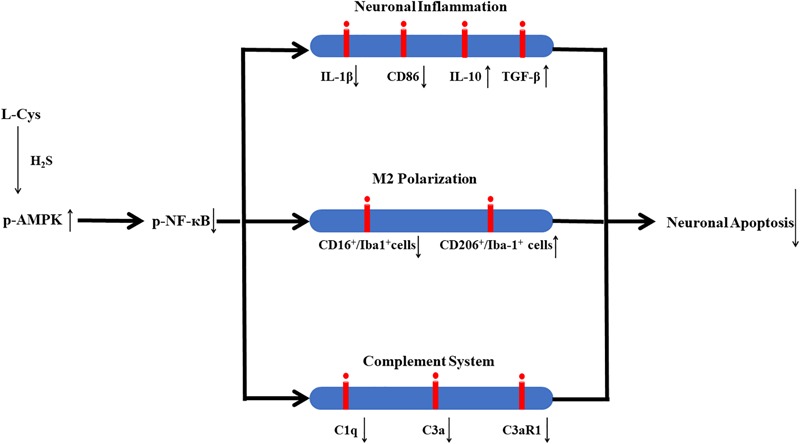
Proposed mechanisms of L-Cys effects on neuronal apoptosis following HI insult. L-Cys-derived H_2_S attenuates neuronal apoptosis after HI in neonatal mice by enhancing microglia M2 polarization and decreasing complement C1q, C3a and C3aR1 expression via activation of the AMPK pathway.

In conclusion, in this study we demonstrate that L-Cys-derived H_2_S attenuates neuronal apoptosis after HI in neonatal mice. This effect of L-Cys involves mechanisms that enhance microglial M2 polarization and decrease complement C1q, C3a and C3aR1 expression via activation of the AMPK pathway (Figure 10). Such findings suggest that L-Cys may serve as a novel candidate for HI treatment.

## Author Contributions

ZW designed the study, interpreted the data, and revised the manuscript. XZ and XC performed the majority of the laboratory work. DX, TL, JQ, and HY performed the animal model. XB performed cell culture. DL designed the manuscript and read and edited the proof. DW involved in study design and revised the manuscript. All authors read and approved the final draft of this manuscript.

## Conflict of Interest Statement

The authors declare that the research was conducted in the absence of any commercial or financial relationships that could be construed as a potential conflict of interest.

## Supplementary Material

FIGURE S1 Schema for L-Cys and AOAA/Compound C treatment schedule, behavioral experiments, and tissue preparation. The pups on postnatal day (P) 7 underwent right common carotid artery ligation, and hypoxia was induced (humidified 8% O_2_ + 92% N_2_ for 1.5 h). L-Cys or AOAA or Compound C (CC) solution was administered via intraperitoneal injection. L-Cys was administered at 1, 2, and 3 days after HI insult. In the HI + L-Cys + CC group and HI + L-Cys + AOAA group, Compound C or AOAA was administered first followed 30 min later with L-Cys injection. The Sham and HI groups were injected identically with the same volume of the vehicle relative to body weight. HI, hypoxia-ischemia; CC, compound C; NORT, novel object recognition test; ip, intraperitoneally; P, postnatal day.

FIGURE S2 AOAA and CC have no effect on the Sham group and HI group. **(A)** Representative brain photographs as determined at 72 h following HI. Arrows indicate sites of significant edema. Brain water content was determined at 72 h following HI insult, *N* = 5 mice/group. **(B)** Representative samples stained with TTC. Infarct volume (white area) was quantified. *N* = 5 mice/group. Values represent the mean ± SD, ^∗∗∗^*p* < 0.001 HI vs. Sham.

## Abbreviations

AMPK: AMP-activated protein kinase
AOAA: aminooxyacetic acid
CBS: cystathionine β synthase
CC: compound C
CD86: cluster of differentiation 86
CM: conditioned media
CNS: central nervous system
DAPI: 4,6-diamidino-2-phenylindole
HBSS: Hanks Balanced Salt Solution
HI: hypoxic-ischemic
H_2_S: hydrogen sulfide
Iba-1: ionized calcium binding adaptor molecule-1
IL: interleukin
JNK: c-Jun N-terminal kinase
NF-κB: nuclear factor κB
PFA: paraformaldehyde
PMSF: phenylmethane-sulfonyl fluoride
RIPA: radio immunoprecipitation assay
RT-PCR: reverse transcription–polymerase chain reaction
TGF-β: transforming growth factor beta
TNF-α: tumor necrosis factor-α
TTC: 2,3,5-triphenyltetrazolium chloride monohydrate
