# Supplementary figures and images for: L-Cysteine-Derived H2S Promotes Microglia M2 Polarization via Activation of the AMPK Pathway in Hypoxia-Ischemic Neonatal Mice
Source: Front Mol Neurosci. 2019 Mar 11;12:58. doi: 10.3389/fnmol.2019.00058 (PMC6421291; doi:10.3389/fnmol.2019.00058)

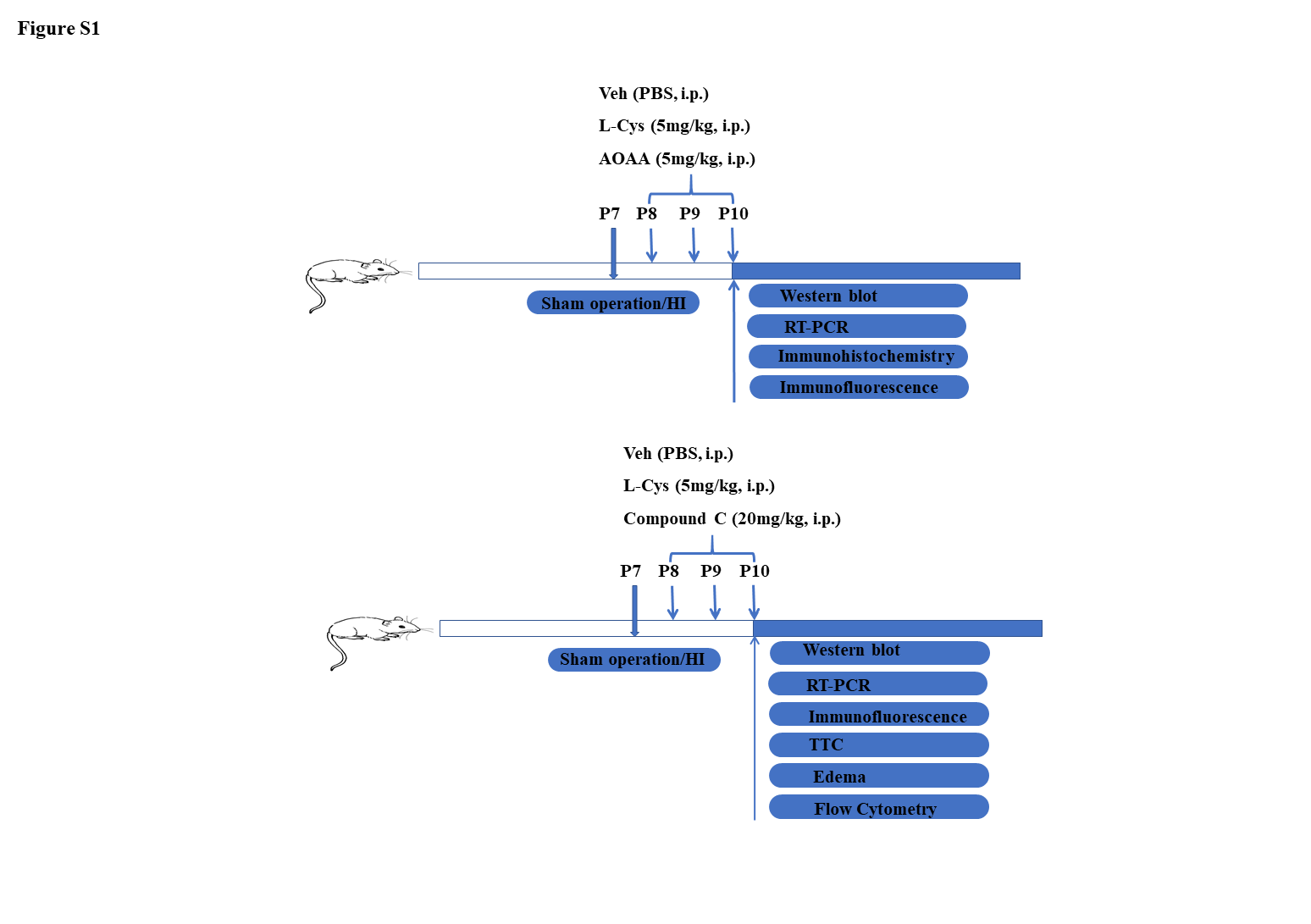

Supplement: FIGURE S1 — Schema for L-Cys and AOAA/Compound C treatment schedule, behavioral experiments, and tissue preparation. The pups on postnatal day (P) 7 underwent right common carotid artery ligation, and hypoxia was induced (humidified 8% O2 + 92% N2 for 1.5 h). L-Cys or AOAA or Compound C (CC) solution was administered via intraperitoneal injection. L-Cys was administered at 1, 2, and 3 days after HI insult. In the HI + L-Cys + CC group and HI + L-Cys + AOAA group, Compound C or AOAA was administered first followed 30 min later with L-Cys injection. The Sham and HI groups were injected identically with the same volume of the vehicle relative to body weight. HI, hypoxia-ischemia; CC, compound C; NORT, novel object recognition test; ip, intraperitoneally; P, postnatal day. [file Image_1.TIF]

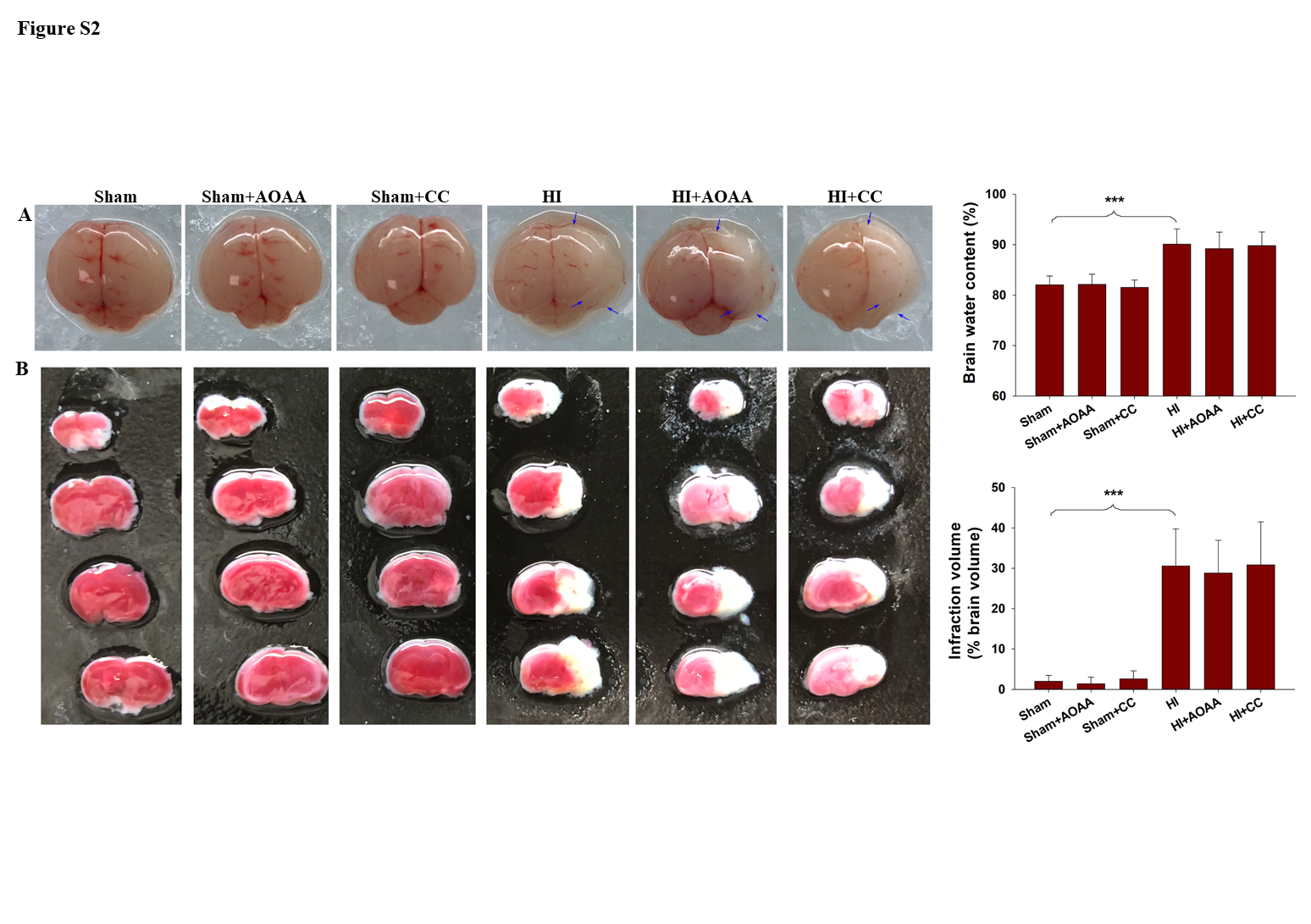

Supplement: FIGURE S2 — AOAA and CC have no effect on the Sham group and HI group. (A) Representative brain photographs as determined at 72 h following HI. Arrows indicate sites of significant edema. Brain water content was determined at 72 h following HI insult, N = 5 mice/group. (B) Representative samples stained with TTC. Infarct volume (white area) was quantified. N = 5 mice/group. Values represent the mean ± SD, ∗∗∗p < 0.001 HI vs. Sham. [file Image_2.TIF]
